# Tir Is Essential for the Recruitment of Tks5 to Enteropathogenic *Escherichia coli* Pedestals

**DOI:** 10.1371/journal.pone.0141871

**Published:** 2015-11-04

**Authors:** Helene H. Jensen, Hans N. Pedersen, Eva Stenkjær, Gitte A. Pedersen, Frédéric H. Login, Lene N. Nejsum

**Affiliations:** Institute of Molecular Biology and Genetics and Interdiciplinary Nanoscience Center, Aarhus University, C. F. Moellers Allé 3, Aarhus, Denmark; Indian Institute of Science, INDIA

## Abstract

Enteropathogenic *Escherichia coli* (EPEC) is a bacterial pathogen that infects the epithelial lining of the small intestine and causes diarrhea. Upon attachment to the intestinal epithelium, EPEC uses a Type III Secretion System to inject its own high affinity receptor Translocated intimin receptor (Tir) into the host cell. Tir facilitates tight adhesion and recruitment of actin-regulating proteins leading to formation of an actin pedestal beneath the infecting bacterium. The pedestal has several similarities with podosomes, which are basolateral actin-rich extensions found in some migrating animal cells. Formation of podosomes is dependent upon the early podosome-specific scavenger protein Tks5, which is involved in actin recruitment. Although Tks5 is expressed in epithelial cells, and podosomes and EPEC pedestals share many components in their structure and mechanism of formation, the potential role of Tks5 in EPEC infections has not been studied. The aim of this study was to determine the subcellular localization of Tks5 in epithelial cells and to investigate if Tks5 is recruited to the EPEC pedestal. In an epithelial MDCK cell line stably expressing Tks5-EGFP, Tks5 localized to actin bundles. Upon infection, EPEC recruited Tks5-EGFP. Tir, but not Tir phosphorylation was essential for the recruitment. Time-lapse microscopy revealed that Tks5-EGFP was recruited instantly upon EPEC attachment to host cells, simultaneously with actin and N-WASp. EPEC infection of cells expressing a ΔPX-Tks5 deletion version of Tks5 showed that EPEC was able to both infect and form pedestals when the PX domain was deleted from Tks5. Future investigations will clarify the role of Tks5 in EPEC infection and pedestal formation.

## Introduction

*Escherichia coli* (*E*. *coli*) are among the most widespread bacterial pathogens that produce diarrhea, and they are one of the leading causes of childhood mortality due to diarrhea in developing countries [1]. A number of different pathogenic strains of *E*. *coli* have been identified, one of the most prevalent being enteropathogenic *E*. *coli* (EPEC) [2].

**Fig 1:**
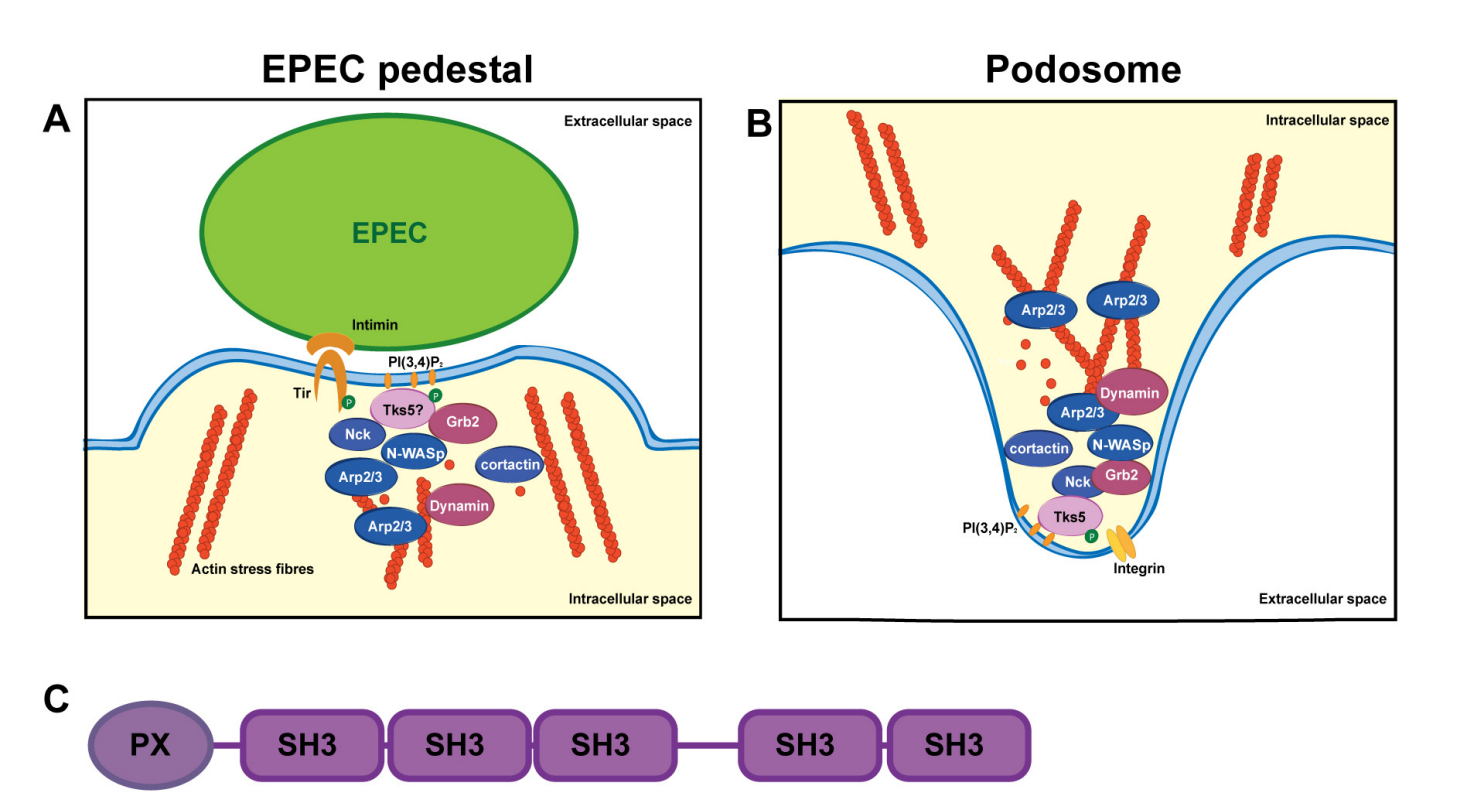
Schematic of central players in actin polymerization in EPEC pedestals and podosomes. A: Upon infection EPEC inserts its own receptor Tir in the host cell membrane. Tir binds intimin, which is inserted into the bacterial membrane. Within the host cell cytosol Tir is phosphorylated by Src and/or Abl/Arg family kinases leading to recruitment of Nck, N-WASp and Arp2/3, which leads to actin polymerization. Also, PI(3,4)P_2_ is synthesized in the membrane. Other proteins, like dynamin and cortactin are also involved in actin polymerization. It is the objective of this study to investigate if Tks5 localizes to the infection site. B: In podosomes signaling by integrins leads to synthesis of PI(3,4)P_2_ and recruitment and phosphorylation of Tks5 and cortactin. Upon binding to PI(3,4)P_2_ Tks5 binds a complex of Nck, Grb2 and N-WASp which again binds Arp2/3 to initiate actin polymerization. Recruitment to or involvement of Tks5 in EPEC pedestal formation is so far unknown. C: Tks5 is composed of one phox homology (PX) domain and five Src Homology 3 (SH3) domains.

Upon entering the gut, EPEC initially adhere to the epithelium of the small intestine through bundle-forming pili (BFP) [3]. EPEC then use the Type III secretion system (T3SS) to translocate various effector proteins into the host cell [4] to modify the cell surface and the underlying cytoskeleton. These types of alterations by pathogenic *E*. *coli* are referred to as attaching and effacing lesions (A/E-lesions) of the intestinal microvilli [5]. One of the T3SS effector proteins, Translocated intimin receptor (Tir), is inserted into the host cell membrane, where it functions as a high affinity receptor for the EPEC adhesin, intimin [6]. Tir-intimin complexes tightly anchor the bacterium to the host cell [6]. In addition, the cytosolic N- and in particular C-terminal regions of Tir are involved in initiating recruitment of a series of proteins, many of which are involved in priming, polymerizing, or regulating actin filament assembly (Fig 1A) [7,8]. This leads to accumulation of filamentous actin at the infection site, creating a pedestal-like structure under the bacterium.

Upon introduction of virulence factors in the host cell, intimin binding triggers the clustering of membrane integrated Tir proteins [9]. Tir is subsequently phosphorylated on tyrosine residue Y474 and to a lesser extent Y454 by host cell kinases of the Src and/or Abl/Arg family [10–12]. Tir phosphorylation leads to recruitment of Nck, N-WASp, and Arp2/3 –three important proteins believed to initiate and facilitate polymerization of actin in the pedestal (Fig 1A) [13,14]. However, other proteins, for example dynamin and cortactin, are also necessary for pedestal formation [15,16], and a number of proteins, for example WIP [17], are believed to influence actin polymerization. Simultaneously, focal adhesion proteins are disassembled by the virulence factor EspC [18,19]. It has been proposed that proteins from focal adhesions including α-actinin [20], talin [21], and vinculin [22] are redirected to the infection site, where they localize in the pedestal. Thus, pedestals may be built from the degraded focal adhesions [19,23]. However, a number of the proteins found in the pedestal, including N-WASp, WIP, and Arp2/3 are not found in focal adhesions [24], indicating that additional mechanisms are involved.

Moreover, it has been shown that phosphoinositol-4,5-diphosphate PI(4,5)P_2_ transiently accumulates at the attachment site in a Tir-dependent manner which in turn promotes EPEC adherence to host cells [25]. Tir also recruits phosphatidylinositol 3-kinase (PI3K) which phosphorylates PI(4,5)P_2_ to form PI(3,4,5)P_3_ [25]. Thereafter, Tir residues Y483 and Y511 initiate recruitment of SH2-containing 5′-inositol phosphatase-2 (SHIP2), which dephosphorylates PI(3,4,5)P_3_ to generate PI(3,4)P_2_ [26]. This phosphorylation-dephosphorylation cascade is involved in regulation of actin polymerization at the attachment site, as disruption of SHIP2 recruitment or expression leads to disordered pedestals [26].

Morphologically and in terms of actin polymerization mechanism, EPEC pedestals resemble podosomes (Fig 1B). Podosomes are small protrusions formed in different types of animal cells, among others myeloid cells, transformed fibroblasts, and epithelial cells [27]; they are involved in cell motility as well as matrix degradation to support cell invasion. Podosomes are formed at pre-existing focal adhesion sites in the basolateral region of the cell, where they can be individual or organized in small circular groups termed “rosettes” [28]. The induction of podosomes leads to the formation of PI(3,4)P_2_ [29], possibly by SHIP2 or synaptojanin2 [30,31]. PI(3,4)P_2_ recruits the scaffolding protein Tyrosine kinase substrate 5 (Tks5), which is phosphorylated by Src [32]. Following phosphorylation, Tks5 binds a multi-protein complex encompassing Nck, N-WASp, and Grb2 [31,33]. However, the composition and assembly of this complex are not fully understood (Fig 1B). Simultaneously, cortactin is recruited and binds a complex of N-WASp and Arp2/3, which is also associated with WIP, Cdc42, and dynamin. These two complexes interact to initiate actin polymerization, which is critical for podosome formation [28]. Later, cortactin coorporates with the Tks5-homologous protein Tks4 in matrix-metalloprotease recruitment and podosome maturation to enable podosomes to invade the extracellular matrix [28,34].

In terms of protein composition, podosomes and pedestals show some major similarities (Compare Fig 1A and 1B). A previous study has suggested that podosome formation is initiated by integrin signaling [35]. During EPEC infection, integrins have also been shown to redistribute to the apical membrane domain in a Tir dependent manner, which is critical for pathogenesis [36]. These observations indicate that like in podosome initiation, the apical recruitment of integrins might be important for pedestal formation. In addition, both pedestals and podosomes are dependent of Src-type phosphorylations, and they both consist of a dynamic actin core with similar changes of phosphoinositide compositions in the membrane [37,38]. Also, both pedestals and podosomes are known to be dependent on Nck, N-WASp, Arp2/3, cortactin, and dynamin to polymerize actin [13,15,16,31,33,38,39]. Moreover, a similar set of additional actin related proteins are observed in both structures, including Grb2, WIP, vinculin, talin, and α-actinin [17,22,40]. Formation of podosomes is also critically dependent on initial recruitment of Tks5 [41], however, no studies have reported whether Tks5 is involved in EPEC pedestal formation. The objective of this study was to characterize the localization of Tks5 in epithelial cells and to investigate whether Tks5 localizes in the EPEC pedestal. Further, the aim was to study if Tks5 is involved in early pedestal formation.

Tks5 was originally identified as a Src substrate [42]. Tks5 contains five SH3 domains, multiple regions that can bind SH2 and SH3 domains, and a PX domain that binds PI(3)P_2_ and PI(3,4)P_2_ [32,42] (Fig 1C). A large number of splice variants of Tks5 have been identified but the splicing pattern as well as the identity and role of these different isoforms remain under debate [43,44]. Phosphorylation of Tks5 is essential for podosome formation [32], and phosphorylated Tks5 acts as a scavenger protein linking phosphoinositides with actin-regulating proteins as discussed above. As Tks5 is expressed in epithelial cells [41,42] and is an initiating factor in podosome formation, Tks5 could potentially also be involved in EPEC infection and pedestal formation (Fig 1A).

To investigate this hypothesis, we generated an epithelial cell line stably expressing Tks5-EGFP. In the cell line, Tks5-EGFP localized to actin bundles. Infection of the cell line revealed that Tks5-EGFP was recruited to the infection site and that Tir was essential for the recruitment. At the infection site, Tks5-EGFP colocalized with actin and N-WASp. Time-lapse microscopy revealed that Tks5-EGFP appeared at the infection site simultaneously with actin and N-WASp within minutes of initial EPEC attachment. Cells expressing a variant of Tks5 without the PX-domain formed pedestals during infection, indicating that this region was not necessary for actin accumulation.

## Materials and Methods

### Bacterial strains, plasmids and antibodies

Enteropathogenic *Eschericia coli* (EPEC) E2348 WT, Δ*tir* [45], Tir_Y474F_ [46], JPN15 [5], and Δ*escN* [47] bacteria of serotype O127:H6 were kindly provided by Dr. Gad Frankel, Imperial College London, UK. EPEC bacteria were grown overnight in LB medium at 37°C. The Tks5-EGFP cDNA construct encoding a human Tks5 variant with NCBI accession number XM_005270295 and murine embryonic fibroblasts [43] were kindly provided by Dr. Sara A. Courtneidge, Sanford-Burnham Medical Research Institute, California. LifeAct-mRuby [48] and N-WASP-mCherry were kindly provided by Dr. Maddy Parsons, King’s College London, UK.

### Cell culture

Madin-Darby canine kidney (MDCK) GII cells [49,50] were cultured in DMEM with low glucose (1 g/L, Gibco). Wild-type (WT) Murine Embryonic Fibroblasts (MEFs) or MEFs with the homozygous ΔPX-Tks5 mutation [43] were routinely cultured in DMEM with high glucose (4.5 g/L, Gibco). Both were supplemented with 10% fetal bovine serum (FBS, Gibco) and 0.5 U/mL penicillin (Sigma), 0.5 g/mL streptomycin (Gibco), 1 mg/mL kanamycin (Gibco) (1xPSK), and maintained in a humidified atmosphere at 37°C and 5% CO_2_. Cells were cultured at up to 80% confluency, and MDCK cells were passaged every 1–4 days; MEFs were passaged every 4–8 days. For experiments, cells were seeded in 22 mm or 35 mm wells with or without collagen coated coverslips.

For transfection, cells were seeded at 50–60% confluency and transfected in suspension with Lipofectamine 2000 (LifeTechnologies) following the manufacturer’s protocol. Transiently transfected cells were transfected two days prior to experiments with GFP-tubulin[51], N-WASp-mCherry, or LifeAct-mRuby. To create a cell line stably expressing Tks5-EGFP, transfected cells were cultured 10 days with 500 μg/mL G418 selection before isolating single clones. Clones were screened by fluorescence microscopy and Western blotting to ensure expression of full length Tks5-EGFP. The resultant cell line was termed Tks5-EGFP-MDCK.

### Infection of MDCK cells

Tks5-EGFP-MDCK cells grown in 35 mm wells were washed three times in 1 mL plain DMEM low to remove antibiotics and dead cells. 1 mL infection medium (DMEM low supplemented with 10% FBS) was added along with 10^8^ EPEC bacteria (based on OD_600_ measurements) from a fresh overnight LB culture. This corresponds approximately to an MOI of 1:500; cells and bacteria were incubated five minutes at 37°C and 5% CO_2_ to allow bacteria to adhere. Then, infection medium and not-attached bacteria were removed, and the cells were washed three times in DMEM low with gentle shaking between each washing step. Cells and attached bacteria were then incubated at 37°C and 5% CO_2_ with 1 mL infection medium for four hours before fixation.

### Immunofluorescent staining and imaging

Cells with or without EPEC infection were fixed for 10 minutes in 4% paraformaldehyde, then permeabilized and blocked in PBS + 0.1% Triton X-100 + 3% bovine serum albumin (BSA) for 5–10 min at RT. For immunofluorescence, coverslips were incubated one hour at RT or overnight at 4°C in primary antibody. The coverslips were then washed three times in PBS before incubating with secondary antibody diluted 1:500 for 1H at RT. Anti-α-tubulin monoclonal primary antibody (sc-32293, Santa Cruz Biotechnology) was diluted at 1:1000 with subsequent labeling with Dylight 594 conjugated secondary antibody (IR-DKMu-003F594, ImmunoReagents, Inc.). To label EPEC bacteria, coverslips were stained with anti-lipid A polyclonal primary antibody (ab20001, Abcam) diluted 1:400 and subsequently in AlexaFluor-647 conjugated secondary antibody (A31571, Invitrogen). For staining of nucleic acids, cells were incubated 30–60 minutes in 2 μg/mL Hoechst. For actin labeling, cells were stained 1H at RT with 0.4 μg/mL phalloidin-rhodamine (Sigma-Aldrich). All stains and antibodies were diluted in PBS + 3% BSA. After staining, coverslips were washed three times in PBS before mounting on microscope slides with Glycergel mounting medium (Dako). Fixed MDCK cells were imaged on a Zeiss LSM 700 confocal microscope system with a 100x objective handled with ZEN software. Individual fluorophores were imaged in separate tracks, and for excitation and emission the used wavelengths were 405/420-800 nm (Hoechst), 488/300-550 nm (Tks5-EGFP), 555/300-630 nm (phalloidin-rhodamine), and 639/644-800 nm (anti-LipidA-Cy5). Laser intensities and gain were adjusted individually for each sample.

### Live microscopy

Cells were grown on collagen coated coverslips and kept in DMEM low without phenol red (Gibco) supplemented with 10% FBS, 25 mM HEPES (Invitrogen), and 1xPSK. For treatment with cytochalasin, 1:1000 cytochalasin from a 1 mg/mL DMSO stock solution was diluted in imaging medium and added directly into the heating chamber.

For live infection experiments, MDCK cells were washed three times in 1 mL DMEM low, mounted in the heating chamber, to which infection medium (DMEM low without phenol red supplemented with 10% FBS and 25 mM HEPES buffer) was added. Cells were infected with 5^.^10^6^ EPEC from an overnight culture or from a sub-culture of EPEC bacteria grown two hours 1:10 in infection medium to prime them for expression of virulent genes.

Live microscopy was performed on a Nikon T*i* ECLIPSE inverted microscope equipped with a Perfect Focus 3 system, a CF160 Apo TIRF 100x objective, an Andor Zyla cMOS camera, and an Oko-Lab heating system kept at 37°C. Imaging was carried out with NIS-Elements software, and image analysis was performed with ImageJ (freely available from NIH [52]).

### Quantification of MEF infection efficiency

WT or ΔPX-Tks5 MEFs grown on collagen coated coverslips in 22 mm wells were washed three times in 1 mL plain DMEM high to remove antibiotics and dead cells. 1 mL infection medium (DMEM high supplemented with 10% FBS) was added along with 4^.^10^7^ WT EPEC bacteria (based on OD_600_ measurements) from a fresh overnight LB culture. Cells and bacteria were incubated five minutes at 37°C and 5% CO_2_ to allow bacteria to adhere. Then, infection medium and not-attached bacteria were removed, and the cells were washed three times in DMEM high with gentle shaking between each washing step. Cells and attached bacteria were then incubated at 37°C and 5% CO_2_ with 1 mL infection medium for six hours before they were fixed and stained with Hoechst and phalloidin-rhodamine as described above. To evaluate infection efficiency, ten random cells of each type were imaged on the Nikon microscope system described above. The number of bacteria per cells was counted, and for each bacterium it was determined whether they formed pedestals based on phalloidin stains. The experiment was performed six times. Outliers with more than 150 bacteria per cell were removed.

### Image processing

Images from confocal and widefield microscopy were processed with ImageJ software [52]. Z-stacks obtained with confocal microscopy were either projected as maximum Z-projections, or single slices were chosen that gave a representative impression of both bacteria and protein localization. For all images, the brightness and contrast were adjusted for the entire image to optimally demonstrate protein localization.

### Western blotting

Equal numbers of WT MDCK and Tks5-EGFP-MDCK cells were seeded in 35 mm dishes and incubated overnight. Medium was removed, and cells were lysed in warm SDS PAGE sample buffer (Bio-Rad) with 20 mM DTT. Proteins were separated on a 7% acrylamide gel and were subsequently transferred onto a PVDF membrane for immunoblotting. Monoclonal anti-α-tubulin antibody (sc-32293, Santa Cruz Biotechnology), and polyclonal anti-Tks5 antibody (09–268, Millipore) were diluted at 1:10,000 and 1:200, respectively. Horseradish peroxidase-conjugated IgG (P0448, Dako) was diluted at 1:5000. Proteins were detected with a chemiluminescence kit (GE Healthcare).

## Results and Discussion

**Fig 2:**
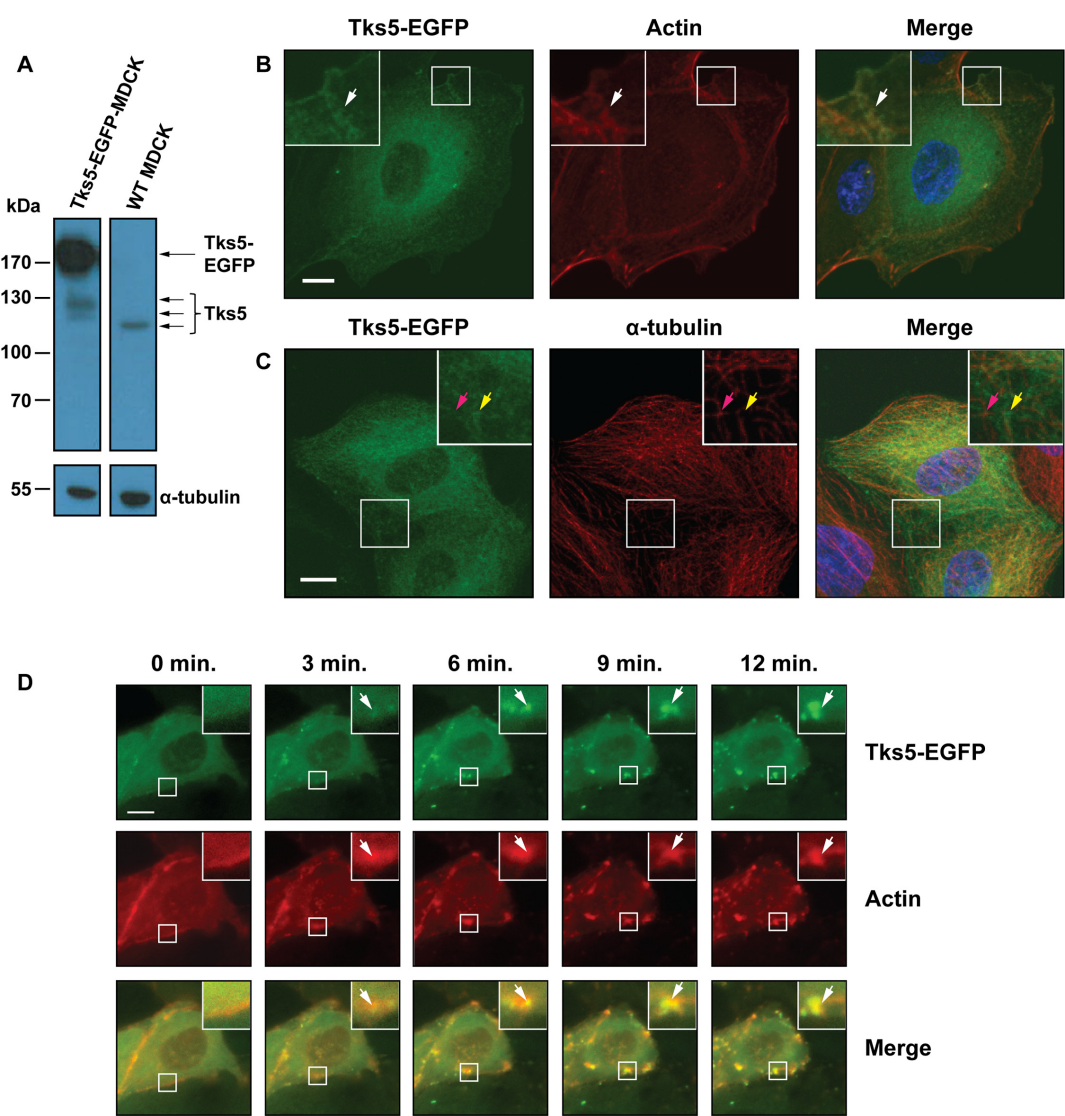
Tks5 localized with bundled actin in MDCK cells. A: Western blot of lysates of Tks5-EGFP-MDCK cells and untransfected MDCK cells probed with anti-Tks5 (top panels) and α-tubulin (bottom panels) antibodies. Arrows indicate bands of Tks5-EGFP and Tks5 isoforms. α-tubulin was included as loading control. One splice variant of Tks5 was observed in WT MDCK cells. Tks5-EGFP-MDCK cells expressed various splice variants of endogenous Tks5 and/or Tks5-EGFP (125–140 kDa); Tks5-EGFP (150–175 kDa) was expressed at a higher level than endogenous Tks5 in the transfected cell line. B and C: Tks5-EGFP-MDCK cells were stained with Hoechst to visualize nuclei (blue) and phalloidin-rhodamine to visualize actin or α-tubulin antibody (red). Images are maximum projections of Z-stacks of 20 or 17 slices, respectively. White arrows in B indicate a site of colocalization of Tks5-EGFP and actin, whereas the yellow arrows in C point to Tks5-EGFP not co-localizing with α-tubulin, and the pink arrows point to α-tubulin. D: Tks5-EGFP-MDCK cells were transiently transfected with LifeAct-mRuby and treated with cytochalasin D to disrupt actin fibers and imaged live with fluorescence microscopy. Timepoints after cytochalasin D addition are indicated. Arrows indicate aggregates of actin, to which Tks5-EGFP also localized. Scale bars correspond to 10 μm.

Enteropathogenic *Escherichia coli* (EPEC) manipulate the host-cell cytoskeleton to form a pedestal, which is an actin-rich structure at the infection site. In several aspects, the EPEC pedestal resembles a podosome, a structure that requires the scavenger protein Tks5 to be formed (Fig 1). However, Tks5 has never been investigated in relation to EPEC infection and/or pedestal formation. To investigate whether Tks5 cell localization was affected by EPEC infection, Madin Darby canine kidney (MDCK) cells were used as a model system. MDCK cells are epithelial cells derived from dog kidney [49,50]. They are non-cancerous and widely used as a model system to study epithelial cells in culture. It is known that epithelial cells express Tks5 [41] but to our knowledge, the subcellular localization of Tks5 in non-cancerous epithelial cells has never been characterized. Thus, the expression levels and localization of Tks5 in MDCK cells were first examined. Western blotting revealed that MDCK cells expressed endogenous Tks5 (Fig 2A, right lane). Unfortunately, it was not possible to detect Tks5 by immunofluorescence using the commercially available antibodies. To overcome this technical limitation, a cell line stably overexpressing Tks5-EGFP was generated (Tks5-EGFP-MDCK) (Fig 2A, 2B and 2C).

Stable expression of Tks5-EGFP in the generated cell line was confirmed by immunoblotting (Fig 2A). Immunoblotting also showed that the stable cells expressed elevated levels and different splice variants or degradation products of Tks5-EGFP and endogenous Tks5 compared to untransfected MDCK cells. Tks5-EGFP was distributed throughout the cell body but also colocalized with filamentous actin at sites of dense actin fibers, and partially at cell-cell junctions and at the periphery of cells (Fig 2B). To test if Tks5-EGFP localization in these regions was actin dependent, time-lapse imaging of cells stably expressing Tks5-EGFP and transiently expressing LifeAct-mRuby to visualize actin filaments was performed during disruption of actin. This revealed that disruption of actin filaments by cytochalasin D also disrupted Tks5-EGFP localization at sites of filamentous actin (Fig 2D). Following disruption, LifeAct-mRuby and Tks5-EGFP colocalized in aggregates, indicating that Tks5-EGFP is directly or indirectly associated with the actin cytoskeleton in MDCK cells.

Immunofluorescence imaging of tubulin in Tks5-EGFP expressing cells revealed that Tks5-EGFP did not co-localize with microtubules in MDCK cells (Fig 2C). Nocodazole treatment to disrupt microtubules did not affect the localization of Tks5-EGFP (data not shown). Therefore, Tks5-EGFP localization in epithelial MDCK cells seemed to be independent of microtubules.

### EPEC recruits Tks5

To study if EPEC recruits Tks5 upon infection, Tks5-EGFP-MDCK cells were infected with EPEC bacteria. Four hours following infection, cells and bacteria were fixed and stained with phalloidin to label the actin cytoskeleton including actin pedestals and Hoechst to label host cell nuclei. Bacterial DNA was also labeled with Hoechst alongside antibodies directed against lipid A, a lipid found in membranes of Gram-negative bacteria. Both Hoechst and lipid A stainings were performed to obtain a more efficient labeling of bacteria.

**Fig 3:**
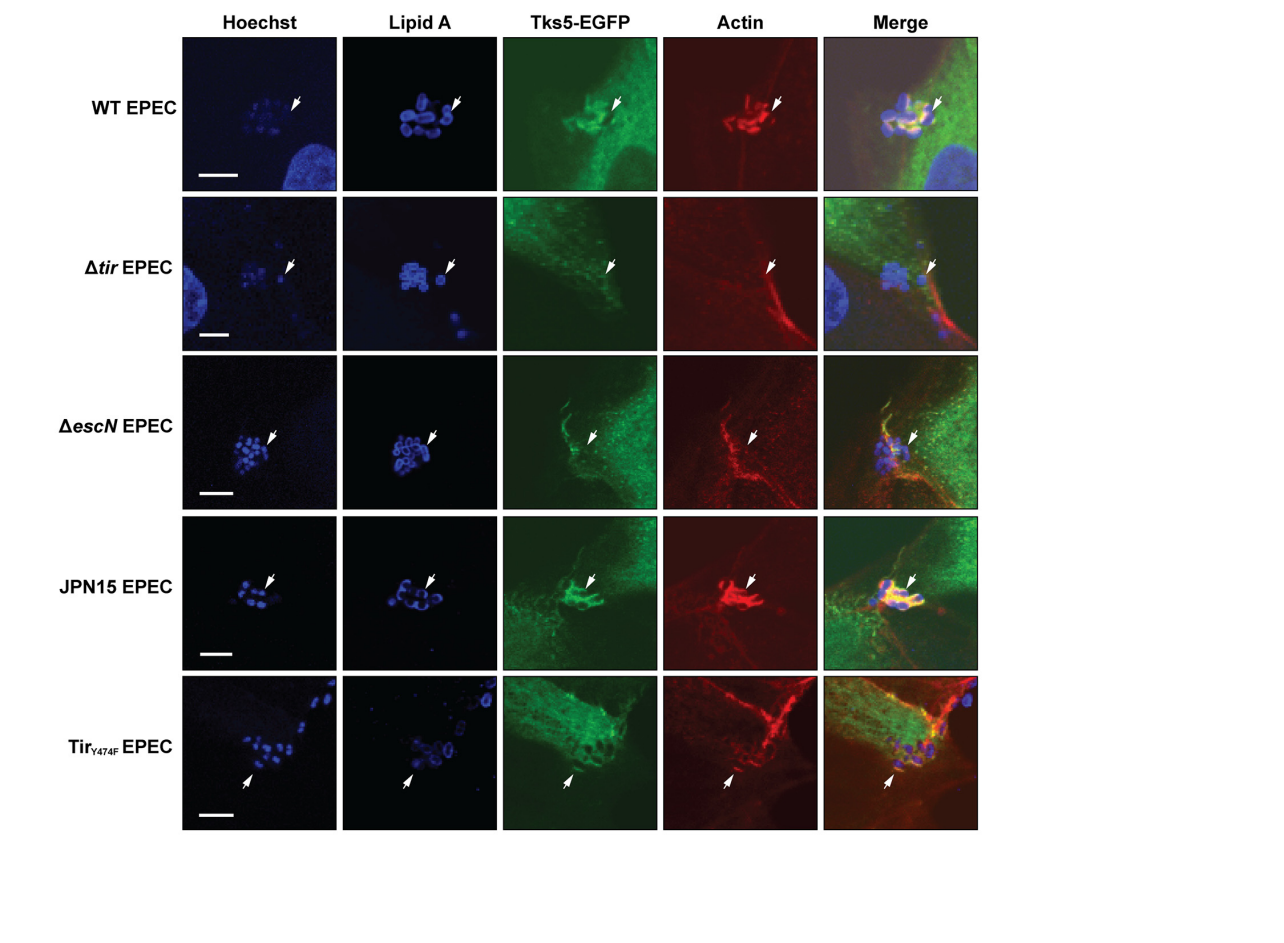
Tks5 localized to EPEC infection site and Tir is essential for the localization. Tks5-EGFP-MDCK cells were infected with WT, Δ*tir*, Δ*escN*, JPN15, or Tir_Y474F_ EPEC for four hours, as indicated. Cells and bacteria were fixed and stained with phalloidin to visualize actin (red) and Hoechst and an antibody directed against lipid A in the bacterial membrane to visualize nuclei and EPEC bacteria (both shown in blue). Z-stacks were acquired on a confocal microscope, and the shown slices were selected to both show EPEC bacteria as well as Tks5-EGFP and actin localization. Tks5-EGFP and actin were recruited to the infection site of WT, Tir_Y474F_, and JPN15 EPEC, where they accumulated around the infecting bacteria. No recruitment was observed for Δ*tir* and Δ*escN* EPEC. Arrows point to the positions of individual bacteria. Scale bars correspond to 5 μm.

As seen in the top panels of Fig 3, WT EPEC bacteria recruited Tks5-EGFP to the infection site, where it colocalized with actin. In contrast, tubulin-GFP was not recruited to the EPEC infection site, revealing that the GFP tag itself could not relocate a cytoplasmic protein to the site of EPEC infection (S1 Fig). It has been reported that several actin-regulating proteins like Grb2, Nck, N-WASp, and Arp2/3 are recruited directly or indirectly to the EPEC infection and that Tir is essential for the recruitment [13,14,22]. To test whether Tir was essential for Tks5 recruitment, Tks5-EGFP-MDCK cells were infected with an EPEC isogenic strain bearing an in-frame deletion of the *tir* gene (Δ*tir*). As shown in Fig 3, the Δ*tir* strain failed to recruit Tks5-EGFP, suggesting that Tir is essential for Tks5 recruitment to the EPEC infection site, either directly or through downstream mediators in the pedestal assembly machinery. Similarly to the Δ*tir* strain, no recruitment was observed when the Δ*escN* mutant was used for infection (Fig 3, third line). The deletion of the T3S-ATPase EscN abolished the secretion of Tir and the other effector proteins via the T3SS. On the other hand, no difference in the recruitment of Tks5-EGFP was observed between the WT and the JPN15 strain, which does not carry the plasmid encoding bundle-forming pili (BFP) (Fig 3, fourth line).

Phosphorylation of tyrosine 474 on Tir is necessary for most of the actin accumulation at the infection site. Y474 phosphorylation recruits Nck, N-WASp, and Arp2/3 [13,22]. However, phosphorylation of Y454 also causes some actin accumulation, although at a much reduced level [53]. The importance of Y474 phosphorylation for Tks5 recruitment was investigated using a strain expressing a Tir variant with the Y474F point mutation (Tir_Y474F_). This variant of Tir cannot be phosphorylated on Y474, leading to a decreased level of actin polymerization at EPEC infection sites. When Tks5-EGFP-MDCK cells were infected with the Tir_Y474F_ strain, Tks5-EGFP localization at the site of infection was affected. Tks5 was rearranged around infecting bacteria in incomplete pseudopedestals along with actin (Fig 3, bottom panels). Thus, despite the inability of Tir_Y474F_ to fully support formation of pedestals, actin and actin associated proteins were recruited. Tks5 may be recruited via other parts of Tir or by actin regulating proteins recruited to the infection site.

Thus, it was observed that Tks5 localized to EPEC pedestals. EPEC strains (Δ*tir* and Δ*escN*) which do not induce pedestal formation did also not recruit Tks5. The Tir_Y474F_ strain which caused actin accumulation with low efficacy also caused low recruitment of Tks5. However, for this strain the recruitment to the pedestal was not clear as there was a low level of actin accumulation. Together, these data demonstrate that the recruitment of Tks5 to the EPEC infection site is T3SS and that Tir is essential for the recruitment.

### Tks5 is recruited instantly and simultaneously with actin and N-WASp

**Fig 4:**
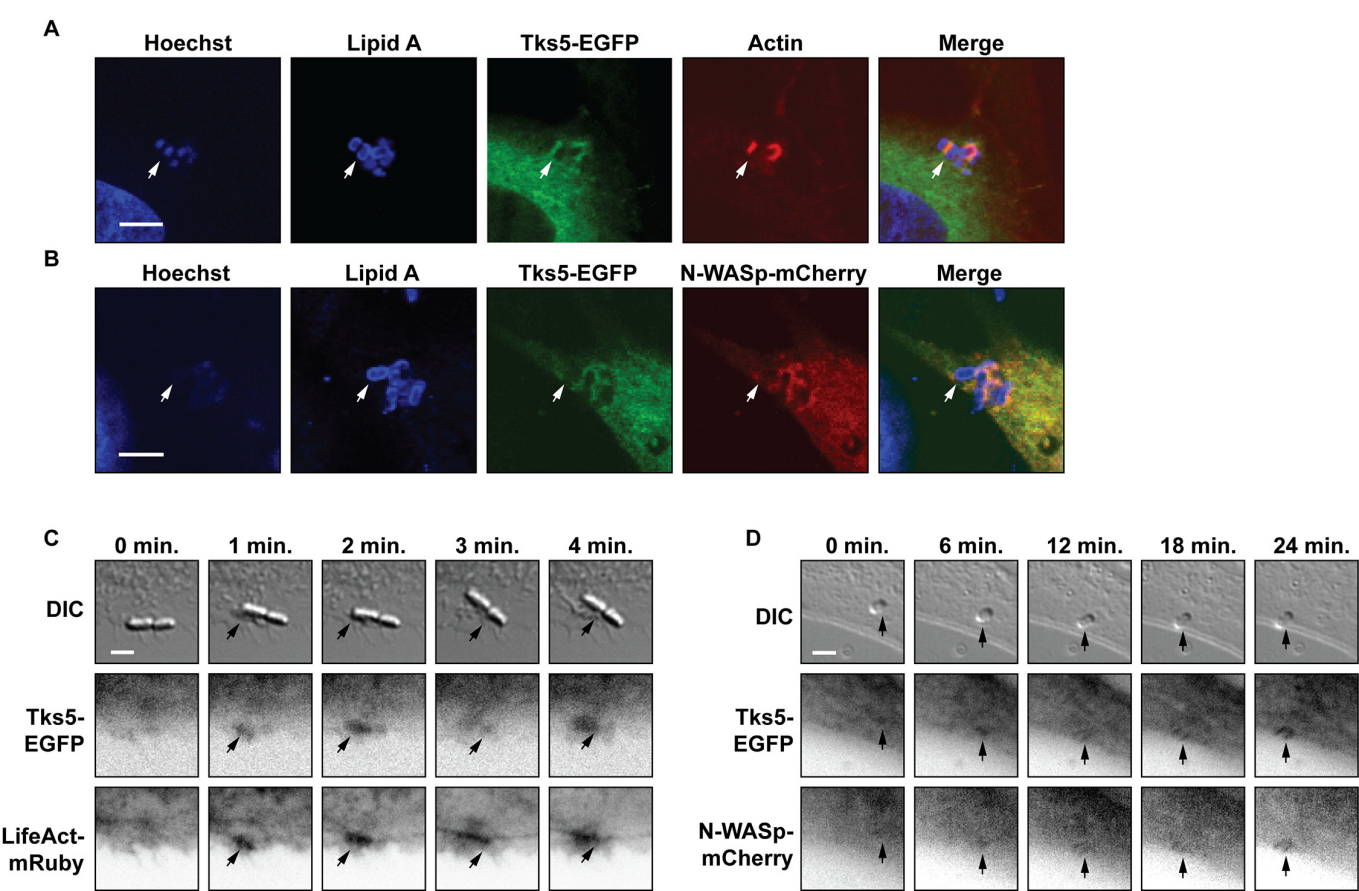
Tks5 was recruited simultaneously with actin and N-WASp to the site of EPEC infection and they colocalized in the pedestal. A: Tks5-EGFP-MDCK cells were infected with WT EPEC for four hours. Cells and bacteria were then fixed and stained with phalloidin to visualize actin (red), Hoechst to visualize DNA and an antibody against lipid A in the bacteria membrane (both in blue). Tks5-EGFP co-localized with actin in the pedestal. B: Tks5-EGFP-MDCK cells transiently transfected with N-WASp-mCherry were infected with WT EPEC for four hours, then fixed and stained with Hoechst and lipid A antibody. Tks5-EGFP co-localized with N-WASp-mCherry at the infection site. Cells and bacteria in A and B were imaged on a confocal microscope, and the shown slices were selected to both show EPEC bacteria as well as protein localization. Arrows point to positions of individual bacteria. Scale bars correspond to 5 μm. C and D: Tks5-EGFP-MDCK cells transiently transfected with LifeAct-mRuby (C) or N-WASp-mCherry (D) were infected with low numbers of WT EPEC bacteria and imaged with widefield microscopy. Tks5-EGFP and LifeAct-mRuby or N-WASp-mCherry are shown as inverted contrast to ease the interpretation. A flash of Tks5-EGFP and LifeAct-mRuby or Tks5-EGFP and N-WASp were observed instantly upon adhesion (exemplified with Tks5-EGFP and LifeAct-mRuby in C). Upon adhesion, Tks5-EGFP was rearranged in the membrane simultaneously with LifeAct-mRuby or N-WASP-mCherry (exemplified with N-WASp-mCherry in D). Arrows point to positions of the initial rearrangements of Tks5 and LifeAct-mRuby or N-WASp-mCherry. Scale bars correspond to 3 μm.

Since Tks5-EGFP was recruited to the EPEC infection site it may play a role in establishment of the pedestal. Unfortunately, generation of full Tks5 knock-out mice has not been successful for others [43], and use of siRNA did not give full knock-down of Tks5 [41]. Thus, the potential involvement of Tks5 in pedestal formation was studied by following the initial phases of infection with time-lapse microscopy. During podosome formation, Tks5 is one of the first effectors recruited [28]. In the initial phase of podosome formation, PI(3,4)P_2_ binds Tks5, followed by the recruitment of Nck, Grb2, and N-WASp to form a complex with Tks5 [31,33]. In EPEC pedestals, Tks5 could serve a similar function during actin polymerization and pedestal formation. If Tks5 is involved in initiation of pedestal formation, Tks5-EGFP accumulation would be expected to occur before or simultaneously with those of actin and N-WASp. Upon infection, Tks5-EGFP colocalized with both actin and N-WASp in EPEC pedestals (Fig 4A and 4B, respectively). Thus, to dissect initial recruitment to forming pedestals, time-lapse microscopy of Tks5-EGFP-MDCK cells transiently transfected with either LifeAct-mRuby to visualize actin or N-WASp-mCherry was performed. The cells were mounted in a heating chamber on a widefield microscope and infected with WT EPEC. Images were captured with DIC and fluorescent light. During development of micro-colonies at the cell surface, recruitment of the fluorescently labeled proteins was imaged. It was observed that the recruitment order was consistent, but the kinetics varied between cells. First, a transient accumulation of Tks5-EGFP and LifeAct-mRuby or Tks5-EGFP and N-WASp-mCherry was detected at the attachment site, as exemplified with Tks5-EGFP and LifeAct-mRuby in Fig 4C. This first, transient accumulation faded and was followed by a slower but robust protein recruitment and rearrangement as exemplified with N-WASp-mCherry and Tks5-EGFP in Fig 4D. In this second phase, Tks5-EGFP, N-WASp-mCherry, and LifeAct-mRuby were localized around individual bacteria and not directly beneath them. Pedestal formation was identified as accumulation of LifeAct-mRuby, N-WASp-mCherry and Tks5-EGFP around individual bacteria.

Thus, EPEC attachment triggered a rapid, but transient recruitment of Tks5-EGFP, LifeAct-mRuby, and N-WASp-mCherry directly under the attaching bacteria at the infection site. This recruitment was followed by an incipient protein rearrangement around the bacteria in the membrane. Importantly, the results presented here indicated that Tks5-EGFP was recruited and rearranged simultaneously with LifeAct-mRuby and N-WASp-mCherry. This would be necessary if Tks5 has a role in priming the EPEC pedestals. However, it cannot be excluded that sequential rearrangements and initiating events occur at kinetics that are above the temporal resolution of the time-lapse microscopy technique used. It is also likely that upon Tir insertion, several different Tir-dependent pathways operate simultaneously in the host cell.

The kinetics of protein rearrangement and pedestal formation upon attachment of EPEC varied between cells. Recruitment in most cells occurred within 2–5 minutes, however in some experiments recruitment occurred gradually in a time-span of 10–20 minutes. Similarly, the timeframe for formation of a defined pedestal varied. However, the approximate time-frame of infection and protein recruitment observed here is in accordance with previously published studies. It has been shown that Tir can be detected inside host cell membranes within 10 minutes post infection [54]. Similarly, actin has been observed to be recruited within the initial 3–4 minutes upon EPEC attachment to the host cell [25]. To our knowledge, there are no available data in the literature describing the kinetics of initial recruitment of N-WASp, Nck, and Arp2/3. However, it has been reported that Tks5 and actin are recruited to sites of PI(3,4)P_2_ generation within 5–10 minutes following initiation of podosome formation [31]. In line with this, Sason *et al*. observed that PI(4,5)P_2_ accumulated at the infection site within 2–5 minutes upon attachment [25].

Time-lapse experiments thus showed that cells respond to bacterial attachment within two to ten minutes as determined by host cell protein rearrangement, which is in accordance with previous studies regarding formation of both pedestals and podosomes. Further, it was observed that Tks5-EGFP was recruited and rearranged at the same time as N-WASp-mCherry and LifeAct-mRuby. Thus, Tks5-EGFP may be recruited to the infection site as a part of the actin polymerization machinery, which instantly starts to rearrange actin.

### The PX domain of Tks5 is not required for infection and pedestal formation

**Fig 5:**
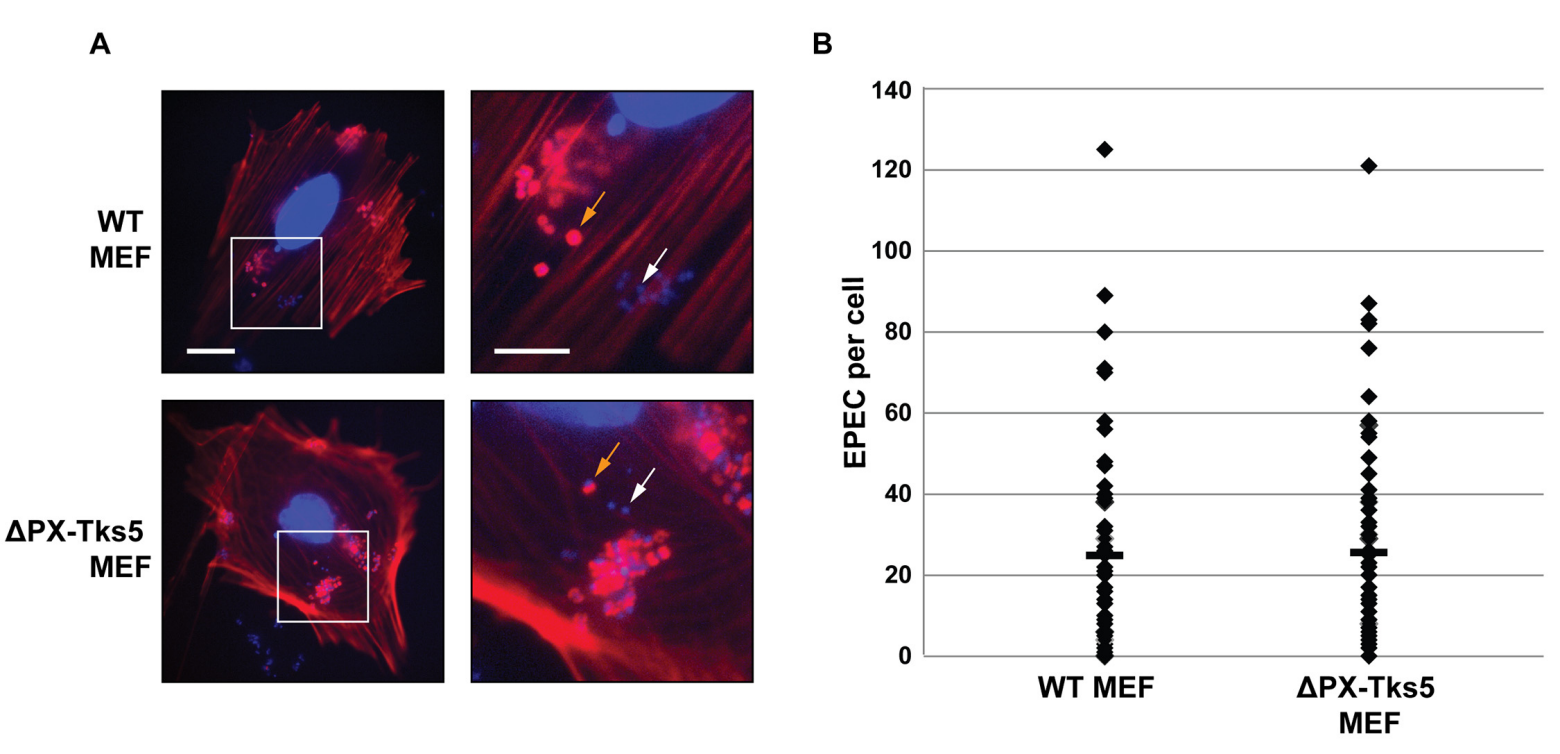
The PX domain of Tks5 is not required for EPEC infection. Murine Embryonic Fibroblasts (MEFs) with wild-type Tks5 expression or expression of the deletion mutant ΔPX-Tks5 only were infected with WT EPEC for six hours. Cells and EPEC were then fixed and stained with Hoechst and phalloidin. A total of 60 cells of each type were imaged, and the number of bacteria infecting each cell was counted, and it was evaluated whether they formed pedestals. Outlier cells with more than 150 infecting EPEC were excluded from the analysis (six cells in total). A: Examples of MEFs infected by EPEC. Orange and white arrows indicate infecting bacteria with and without pedestal formation, respectively. Scale bars correspond to 20 μm for whole-cell images and 10 μm for inserts. Colors were adjusted to optimally visualize infecting bacteria. B: Number of infecting EPEC per cell with the average numbers indicated by a bar.

As Tks5 in podosomes bind PI(3,4)P_2_ to initiate podosome formation, we hypothesizedthat the interaction between Tks5 and PI(3,4)P_2_ was necessary for pedestal formation. It has been shown that PI(3,4)P_2_ is synthesized in the membrane at the infection site [26], and Tks5 binds these *via* the PX domain [55]. To address the potential role of the PX domain a Murine Embryonic Fibroblast (MEF) cell line only expressing Tks5 isoforms lacking the PX domain (ΔPX-Tks5) was used [43]. WT MEFs and ΔPX-Tks5 MEFs were infected for six hours with equal numbers of WT EPEC, fixed and stained, and the number of infecting bacteria and their ability to form pedestals were evaluated. The number of EPEC bacteria infecting 59 WT MEFs and 56 ΔPX-Tks5 MEFs were 1414 and 1379, respectively (Fig 5A). As shown in Fig 5B the average number of infecting bacteria per cell was similar, however with high variance: 24.1 bacteria per WT MEF, and 27.8 bacteria per ΔPX-Tks5 MEF. Pedestals were observed at the infection site of 82.8% of bacteria infecting WT MEFs and 86.5% of bacteria infecting ΔPX-Tks5 MEFs. Thus, the infection efficiency on MEFs was not affected by the PX domain deletion; hence this domain of Tks5 is not essential for pedestal formation.

Tks5 harbors five SH3 domains [42], which have many potential interaction partners. Since Tir was essential for Tks5 recruitment we hypothesized that Tks5 and Tir could interact in the pedestal. *In silico* analysis of Tir using SH3PepInt software [56] predicted two putative binding sites (N_11_GNHLIPPAPPLPSQ_25_ and V_61_DSRDIPGLPTNPSR_75_) for SH3 domain containing proteins such as SRC or nephrocystin-1. Neither a full Tks5 knock-out nor a mutant expressing only ΔSH3 Tks5 were available to further address the necessity of Tks5 SH3 domains in pedestal formation. Therefore, to verify this hypothesis, we used a pull down approach where a C-terminal His tagged Tir was used as bait. Unfortunately, neither the endogenous Tks5 nor Tks5-EGFP from a Tks5-EGFP-MDCK cell lysate specifically bound to Tir (data not shown). Thus, our results from the pull down experiment did not support a Tir-Tks5 interaction, and it could therefore not be determined whether Tks5 was recruited directly by Tir or by downstream mediators of Tir. It is however plausible that the Tir-Tks5 interaction occurs transiently and requires other protein partners or specific membrane lipids.

## Conclusions

Formation of the EPEC pedestal has been subjected to intensive studies, and numerous proteins have been observed to localize to the forming pedestal upon EPEC attachment. The functions of several of the host proteins involved in pedestal formation have been determined; however, the chronology of recruitment and interactions still remain elusive. The present study shows that in epithelial cells, the podosome-specific scavenger protein Tks5 localized with bundled actin. We showed an early recruitment of Tks5 to the infection site of attached EPEC, and that Tir is essential for the recruitment. Moreover, Tks5, N-WASp and actin were simultaneously recruited to the site of initial attachment as well as to the forming actin pedestal. The PX domain was not essential for pedestal formation, and an interaction between the SH3 domains of Tks5 and Tir could not be determined. Therefore, further experiments are needed to address whether Tks5 is recruited directly by Tir or if other proteins or lipids are involved in the molecular mechanisms leading to recruitment.

## Supporting Information

S1 Fig Tubulin-GFP was not recruited to the EPEC infection site. Tks5-EGFP-MDCK cells or WT MDCK cells transiently expressing GFP-tubulin were infected with WT EPEC for four hours. Cells and bacteria were fixed and stained with Hoechst to visualize DNA and phalloidin to visualize actin. The samples were imaged on a confocal microscope, and slices from a Z stack were chosen to show localization of the tagged proteins in the infection area. Arrows point to examples of EPEC pedestals. Scale bar corresponds to 3 μsm.
(TIF)
