# Supplementary figures and images for: Tir Is Essential for the Recruitment of Tks5 to Enteropathogenic Escherichia coli Pedestals
Source: PLoS One. 2015 Nov 4;10(11):e0141871. doi: 10.1371/journal.pone.0141871 (PMC4633291; doi:10.1371/journal.pone.0141871)

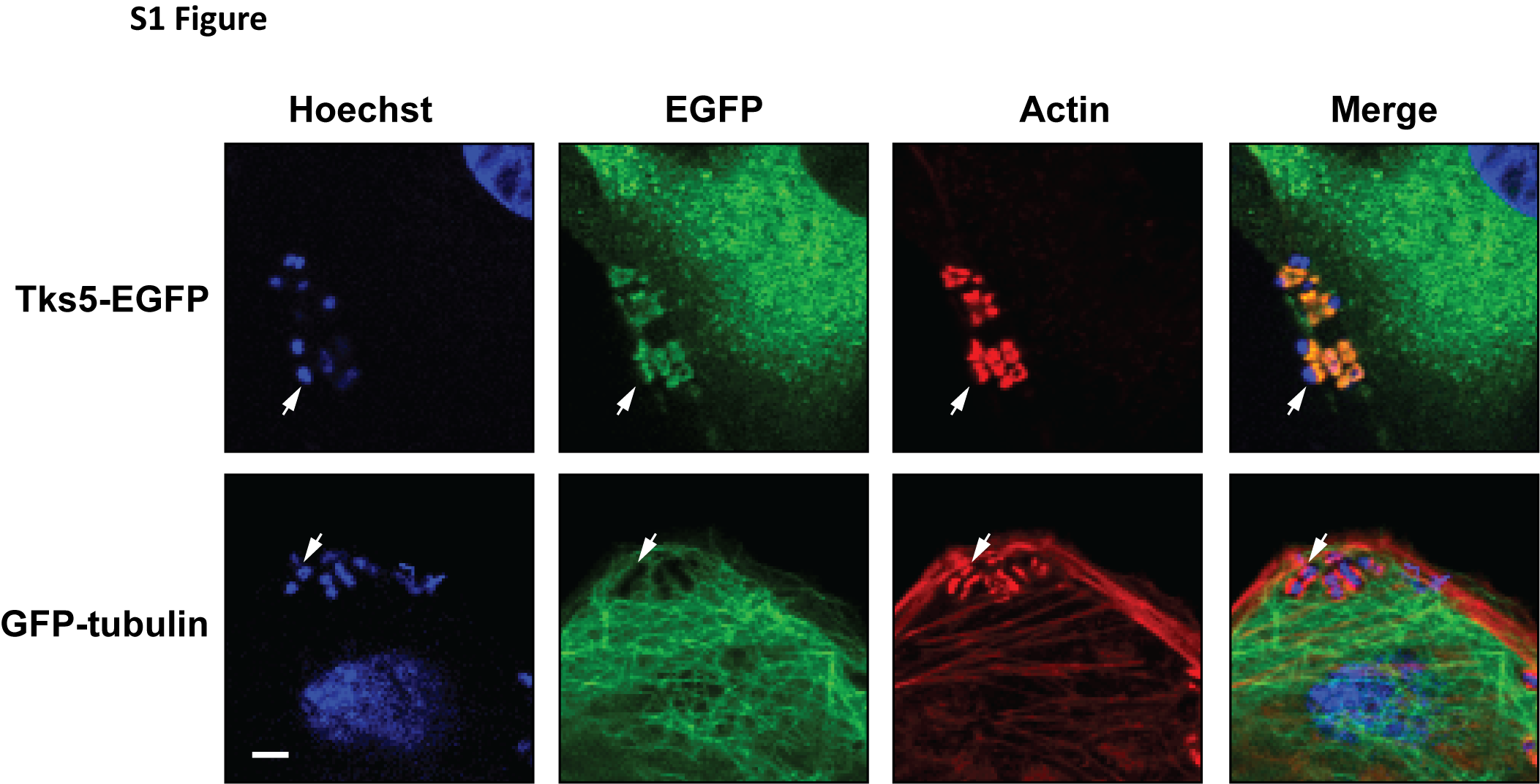

Supplement: S1 Fig — Tks5-EGFP-MDCK cells or WT MDCK cells transiently expressing GFP-tubulin were infected with WT EPEC for four hours. Cells and bacteria were fixed and stained with Hoechst to visualize DNA and phalloidin to visualize actin. The samples were imaged on a confocal microscope, and slices from a Z stack were chosen to show localization of the tagged proteins in the infection area. Arrows point to examples of EPEC pedestals. Scale bar corresponds to 3 μsm. (TIF) [file pone.0141871.s001.tif]
